# An anomalous band originating from the fabella causing semimembranosus impingement presenting as knee pain: a case report

**DOI:** 10.1186/s13256-018-1946-y

**Published:** 2019-01-09

**Authors:** V. Adukia, M. Ricks, K. Colquhoun, N. Flynn

**Affiliations:** 1 Trauma and Orthopaedic Department, Queen Alexandra Hospital, Portsmouth, UK; 2 Trauma and Orthopaedic Department, University Hospital Southampton, Southampton, UK

**Keywords:** Knee, Pain, Fabella, Aberrant, Semimembranosus

## Abstract

**Background:**

The presentation of patients with knee injuries which can have a significant impact on their ability to work and perform activities of daily living is constantly rising. The posterolateral corner of the knee has a complex anatomy of muscles, tendons, and ligaments, with huge variation in the population. The fabella is one such structure, found in the posterolateral corner of the knee, which can serve as a common origin point of various ligaments.

**Case report:**

We present a case report of a 53-year-old white man who presented with atraumatic, posterior knee pain and was found to have a congenital, anomalous band originating from the fabella, causing semimembranosus impingement. This was diagnosed with magnetic resonance imaging; he underwent division of the anomalous band, which resulted in complete resolution of his symptoms.

**Conclusion:**

We propose that patients who present with posterior knee pain, without any history of trauma, and have no abnormalities on plain radiographs, should undergo magnetic resonance imaging of their knees. This will help in assessing the ligament complex in the posterior compartment of the knee, and exclude impingement of the semimembranosus as an, albeit rare, cause of posteromedial knee pain.

## Background

The presentation of patients with knee injuries which can have a significant impact on their ability to work and perform activities of daily living is constantly rising [1]. Posterolateral corner injuries can be associated with ligamentous rupture of the anterior or posterior cruciate ligaments. In the absence of a cruciate injury, posterolateral corner injuries can be easily missed [2]. The posterolateral corner has a complex layered structure of muscles, tendons, and ligaments, which are vital to the stability and function of the knee [1, 3]. The fabella is a sesamoid bone, usually found in the muscular fibers of the lateral head of gastrocnemius in the posterolateral corner. Although there is a huge variation in its incidence within the human population [4], it has been shown to be present in 30 [1] to 68.6% of the population [5]. Presence of the fabella itself can cause symptoms such as posterolateral knee pain and common peroneal palsy that worsens with knee extension. This is thought to be due to the fabella impinging on the lateral femoral condyle and/or the common peroneal nerve, and is termed “fabella syndrome” [5, 6].

The fabella has been noted to serve as a common origin point of ligaments, such as the oblique popliteal, arcuate, and fabellofibular ligaments, which function as a complex unit to add to the stability of the posterior compartment of the knee [6–9]. Unfortunately, there is often a huge variation seen in the anatomy of these ligaments. Minowa *et al.* [7] conducted cadaveric studies which demonstrated not only this variation in anatomy, but also the presence of anomalous native tissues “connecting the different connective tissue planes.”

We present a case report of an anomalous band originating from the fabella causing semimembranosus impingement. To the best of our knowledge, there have been no case reports of these aberrant tissues causing symptomatic knee pain.

## Case presentation

A 53-year-old white man presented to our knee clinic with knee pain. The pain was located in the posteromedial aspect of his left knee and first presented whilst training for a marathon. The pain was a continuous dull ache, which would often wake him from sleep. He had no improvement from conservative management trialled by his general practitioner, which included rest, ice, elevation, orally administered non-steroidal anti-inflammatory drugs, and physiotherapy. There was no history of trauma, locking, or giving way of the knee. He was otherwise fit and well with no medical co-morbidities; he was very active and had not had any previous injuries or surgeries to his left knee.

A physical examination revealed normal alignment of his knee and hindfoot, no effusion, and an area of point tenderness posteromedially, not over the hamstrings or the pes anserinus. There was full range of movement with a positive medial step off and good tracking of the patella with no gross patellofemoral crepitus. He also did not have any significant ligamentous instability and an examination of his ipsilateral hip joint was normal.

**Fig. 1:**
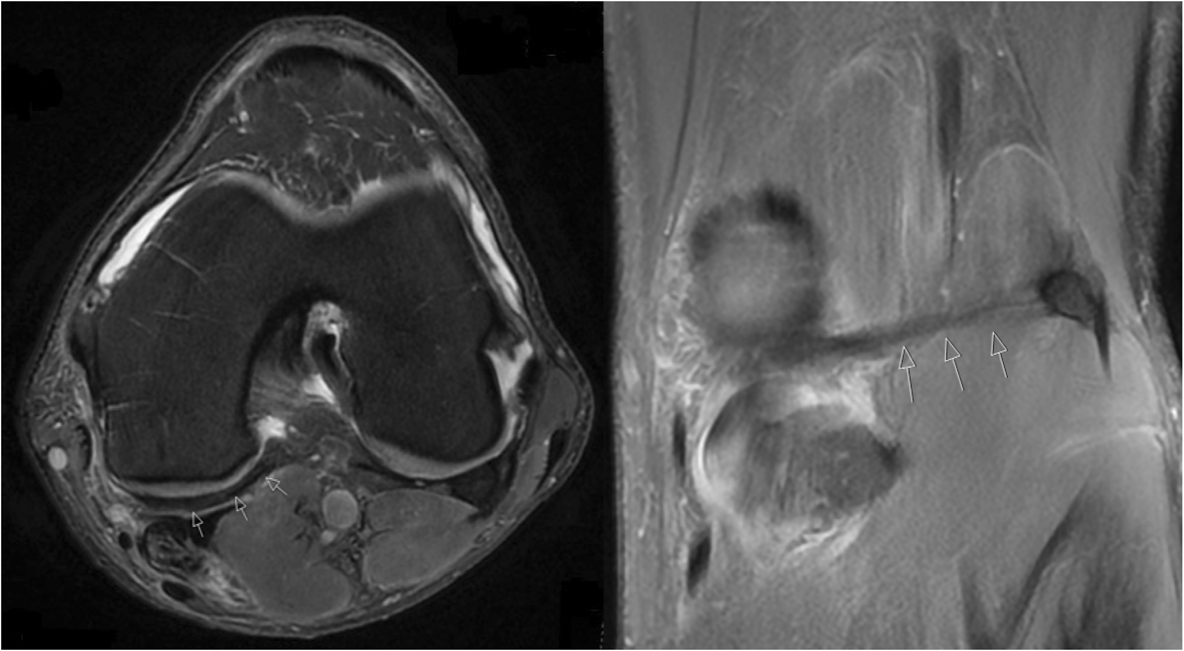
Axial and coronal magnetic resonance imaging images of the right knee, depicting the cord-like structure, originating from the fabella (*arrowed*)

**Fig. 2:**
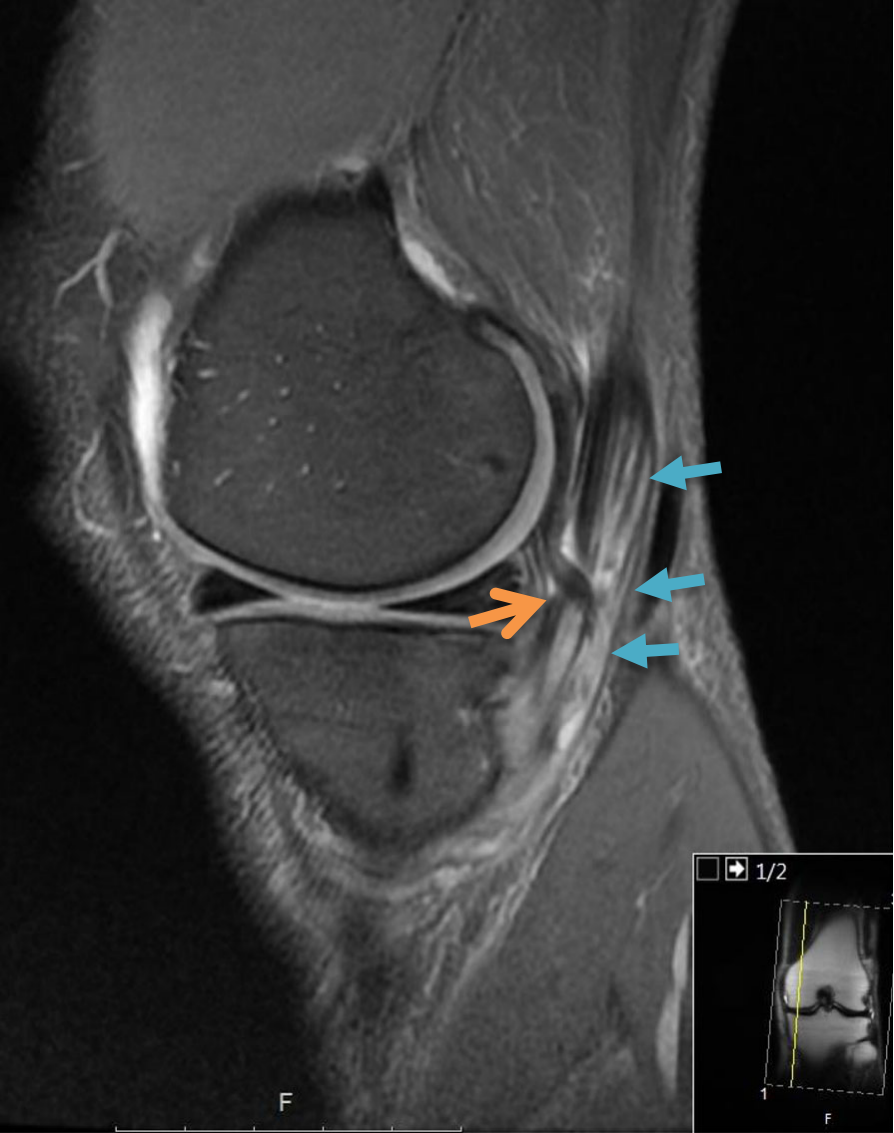
Sagittal magnetic resonance imaging of the right knee, depicting the cord-like structure (*orange arrow*), originating from the fabella, impinging on the semimembranosus tendon (*blue arrows*)

Plain radiographs taken at the time of presentation did not reveal any significant abnormalities and magnetic resonance imaging (MRI) was organized, which demonstrated the presence of a cord-like structure that originated from the fabella and passed medially, dividing into two parts around the semimembranosus tendon (Fig. 1). The superficial part appeared to blend in with the semimembranosus tendon sheath itself, whereas the deeper part was thought to blend in with the superficial fascia of the gracilis and semitendinosus. This was associated with the presence of diffuse thickening of the distal semimembranosus tendon suggesting impingement of the tendon (Fig. 2).

**Fig. 3:**
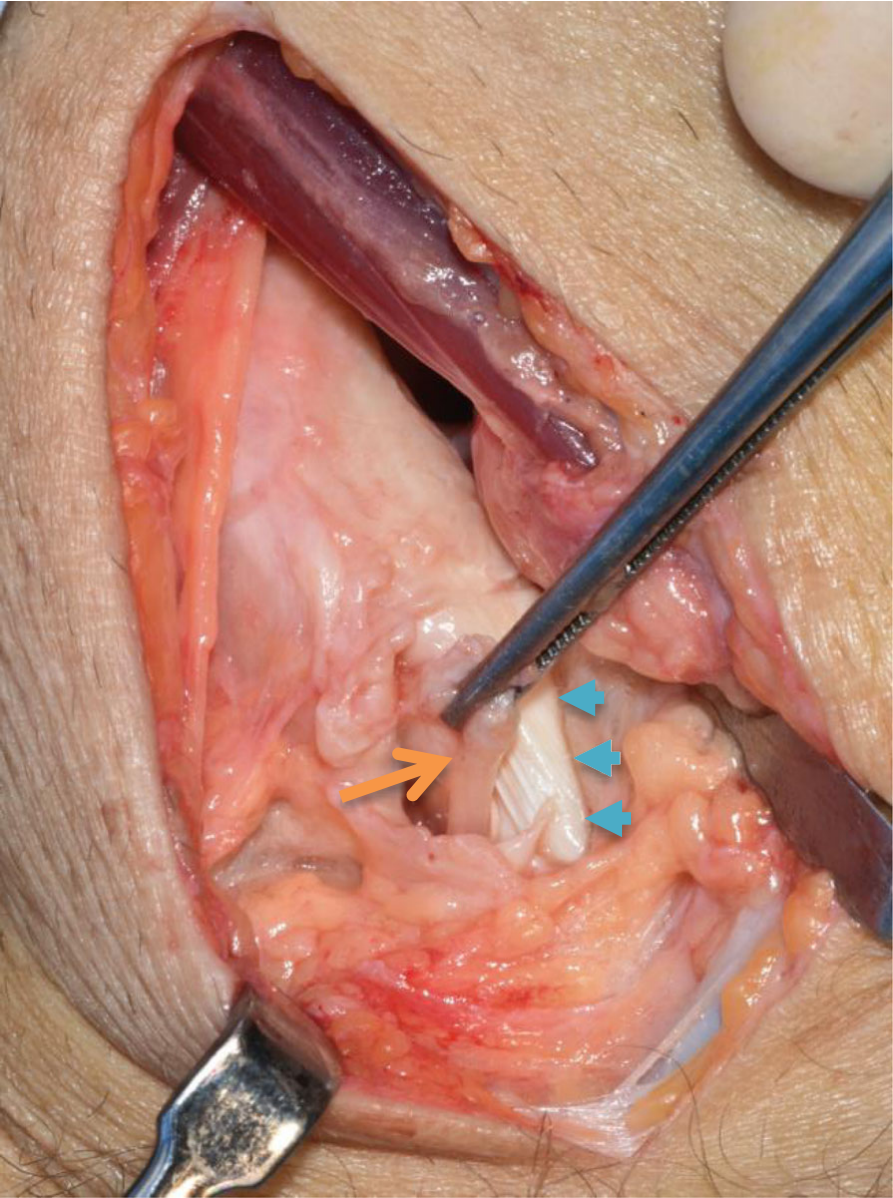
Image taken at the time of surgery, depicting the cord-like structure (*orange arrow*) after it had been divided. The semimembranosus tendon can be seen directly underneath it (*blue arrows*)

As he continued to be symptomatic, and conservative measures had failed, he underwent a knee arthroscopy which demonstrated a grossly thickened semimembranosus with fluid collection around it. A band arising from the fabella, running transversely across the popliteal fossa and around the semimembranosus tendon was noted, confirming the diagnosis of semimembranosus impingement. This band, thought to be congenital in nature, was divided, and the semimembranosus fully released (Fig. 3).

Postoperatively, he recovered well and was allowed to fully weight bear with crutches. He was followed up at 6 weeks post-surgery, at which time his symptoms had resolved and he was back to training for a marathon.

## Discussion and conclusion

This patient had a congenital band passing from his fabella, running transversely across the popliteal fossa and blending in with the fibers of semimembranosus, semitendinosus, and gracilis. Due to his highly active lifestyle, he sustained minor trauma to his knee, which induced fibrosis of the band, leading to impingement of the semimembranosus tendon.

A MRI was vital in reaching a diagnosis and helped delineate the anatomy of the posterior compartment of his knee. Whereas abnormal thickening of the semimembranosus is a relatively common finding on MRI, especially in elderly patients with a history of trauma or osteoarthritis [10], constriction of the semimembranosus due to a cord-like band has not been noted before. This diagnosis was confirmed at the time of the knee arthroscopy and the band divided to release the semimembranosus, resulting in complete resolution of our patient’s symptoms.

This is the first case study reporting the presence of an anomalous band, arising from the fabella, constricting the semimembranosus tendon and leading to pain in the posterior compartment of the knee. We propose that patients who present with posterior knee pain without any history of trauma and who have no abnormalities on plain radiographs undergo an MRI of their knees. The MRI can be used to assess the ligament complex in the posterior compartment of the knee and to exclude impingement of the semimembranosus as an, albeit rare, cause of posteromedial knee pain.

## Abbreviation

MRI: Magnetic resonance imaging
